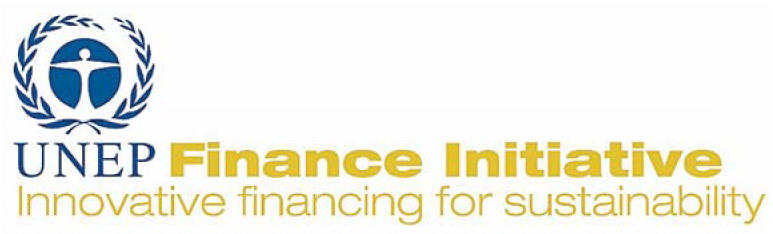# EHPnet: UNEP Finance Initiative

**Published:** 2006-08

**Authors:** Erin E. Dooley

Around the time of the Rio Earth Summit, the UN Environment Programme began raising awareness of environmental and sustainability issues among the financial industry. Today, the program’s Finance Initiative (UNEP FI) helps more than 160 firms—including some of the world’s largest banks, insurers, and fund managers—to integrate sound environmental and sustainability practices into their operations. The UNEP FI website, located at **http://www.unepfi.org/**, provides an in-depth look at the organization’s activities.

Signatories commit to upholding the principles outlined in one of the two UNEP FI statements of principles (one is specifically for financial institutions, the other for insurers). These voluntary, non-binding statements reflect the belief that sustainability is not just a responsibility but also a sound business practice. Each signatory pays an annual fee, attends UNEP FI General Meetings, and submits a brief annual report on steps the institution has taken that year to advance its commitment to the relevant UNEP FI statement. Signatories may also participate in training and workshops, task force meetings, global roundtables, and themed conferences sponsored by the initiative. The Our Signatories section of the website lists the UNEP FI signatories and includes the text of the two UNEP FI statements of principles.

The UNEP FI sponsors regional activities, work groups that focus on finding creative ways to link finance and sustainability, training programs, and research. The Work Programme section of the website describes the core activities that the UNEP FI focuses on. The Climate Change Working Group examines carbon finance (which includes the use of tradeable “carbon credits”), policy and regulation debates, and renewable energy. The UNEP FI is also conducting four projects related to finding ways to link social, environmental, and governance issues with responsible investment practices. A third core activity is sustainability management and reporting, the development of environment and social performance indicators specially tailored to the finance industry. The UNEP FI is also exploring ways to invest responsibly in politically risky nations, and to leverage water-related issues to the benefit of both resource sustainability and business.

The Regional Activities section details the work of task forces that the UNEP FI has established in the African, Asian/Pacific, Central/Eastern European, Latin American, and North American regions. These task forces are responsible for overseeing UNEP FI activities of local signatories and for facilitating relationships among signatories that allow them to interact and share information.

Visitors to the site can also sign up to receive the UNEP FI e-bulletin, which contains a rundown of news, events, and new publications. Back issues of the e-bulletin are available, as are all issues of the quarterly UNEP FI newsletter, *0.618...* (the name refers to the golden ratio and reflects the ratio of risk to reward inherent in sustainable development). This newsletter features articles written by experts in the field.

## Figures and Tables

**Figure:**